# Management of acutely injured cattle by on farm emergency slaughter: Survey of veterinarian views

**DOI:** 10.3389/fvets.2022.976595

**Published:** 2022-11-10

**Authors:** Paul McDermott, Aideen McKevitt, Flávia H. Santos, Alison Hanlon

**Affiliations:** ^1^School of Veterinary Medicine, Veterinary Sciences Centre, University College Dublin, Dublin, Ireland; ^2^Veterinary Department, Department of Environment, Climate Change, and Agriculture, Mayo County Council, Castlebar, Ireland; ^3^School of Agriculture and Food Science, University College Dublin, Dublin, Ireland; ^4^School of Psychology, University College Dublin, Dublin, Ireland

**Keywords:** on-farm emergency slaughter, casualty slaughter, euthanasia, veterinarians, perceptions, acute injury

## Abstract

**Background:**

Fitness to transport is a key provision in animal welfare regulations in the European Union, and for the management of acutely injured cattle. Whilst treatment may be appropriate for some injuries, three common production outcomes for acutely injured cattle are; on farm emergency slaughter (OFES), casualty slaughter (CS) or euthanasia. The aims of this study were to evaluate the perceptions of veterinarians, working in Ireland, on the use of OFES for the management of acutely injured cattle and to evaluate the influence of capacity, willingness and opportunity on their ability to operate OFES.

**Methodology:**

Two online surveys of veterinarians working in Ireland, Private Veterinary Practitioners (PVPs) and Official Veterinarians (OVs), were conducted through Qualtrics^XM^ over a 7-week period between April and June of 2021. Quantitative and qualitative questions were developed and analyzed using the tripartite framework of capacity, willingness, and opportunity to collect relevant data about the management of acutely injured cattle and the provision of OFES in Ireland by veterinarians.

**Results:**

43 OVs and 85 PVPs participated in the survey. OVs regulated on average 4.2 abattoirs, of which 21.6% accepted OFES. Participants reported 343 and 377 OFES and CS, respectively, in 2020. 62.4% PVPs had not certified cattle for OFES, or CS. Limb fracture accounted for 79% OFES, 34.5% CS and 47.9% euthanized acutely injured cattle. 63.3% OVs and 44% PVPs were not aware of abattoirs providing OFES within 100 km of their workplace. Lack of availability of OFES negatively associated with PVP knowledge of the procedure. Regulations and guidelines were the most common source of information on OFES for PVPs.

**Conclusion:**

Increasing the availability of OFES may help to improve the management of acutely injured cattle, especially those with limb fractures that are unfit for transport.

## Introduction

On farm emergency slaughter (OFES) “*is the slaughter outside the slaughterhouse, of an otherwise healthy animal, which has suffered an accident that, for welfare reasons, prevented its transport to a slaughterhouse”* (1). The procedure is designed to prevent the transport of acutely injured cattle to abattoirs for Casualty Slaughter (CS) and provides an alternative to the euthanasia and disposal of cattle that are otherwise fit for human consumption. CS, on the other hand, is “*the slaughter at an abattoir* of *injured cattle that have been deemed fit for transport under veterinary certification*” (1).

In May 2005, delegates to the World Assembly of the World Organization for Animal Health (WOAH, previously OIE), which represents 180 Member Countries and Territories, adopted ten animal welfare standards into their Terrestrial Code. Some of these standards are deemed to assess the degree of impaired functioning associated with injury, disease, and malnutrition of animals (2). In Article 7.5.1 of the OIE Terrestrial Animal Health Code, it is stated “*that the need to ensure welfare of food animals during pre-slaughter and slaughter processes until they are dead in the slaughterhouses. Animals slaughtered outside of slaughterhouses should be managed to ensure their transport, lairage, restraint and slaughter is carried out without causing undue stress to the animals”* (3). In the European Union (EU) the main regulations pertaining to the transport and slaughter of cattle is Council Regulation (EC) No. 1/2005 of 22 December 2004 on the protection of animals during transport and related operations (4). It states “*no person shall transport animals or cause animals to be transported in a way likely to cause injury or undue suffering to them”* (4).

The management of acutely injured cattle by OFES is well established in certain parts of Ireland. Legal provision for OFES was introduced in Ireland in 2009, to provide an outlet for acutely injured livestock and reduce the prevalence of CS of cattle that were deemed unfit for transport (5). A study conducted in the Republic of Ireland between 2006 and 2008, demonstrated that, of cattle consigned under veterinary certification to four large abattoirs, over 60% of the cattle could have been designated for OFES, if the procedure was available (5). Veterinarians are faced with significant conflicts of interest when issuing certificates for the transport and slaughter of acutely injured cattle, which was reported as one of the three most important ethical challenges faced by veterinarians in Ireland (6). The procedure of OFES requires the agreement of key stakeholders namely abattoir owners, Official Veterinarians (OVs), Private Veterinary Practitioners (PVPs) and farmers (1). Furthermore, it requires ante-mortem inspection at the farm by the PVP and post-mortem examination by the OV and relevant certification to verify veterinary public health requirements.

In the absence of OFES, acutely injured cattle that are deemed unfit for transport may be treated or euthanised depending on the severity of the injury. The national registration system for cattle in Ireland (Animal Identification and Movements System; AIMS) records on-farm mortality, which may provide an approximation of the number of animals euthanized on-farm. For example, in 2020 there were 228,527 on farm deaths which represents 3.5% of the total cattle population (7). A study conducted in 2015 showed that in Northern Ireland 0.11 % (*n* = 3,657) of bovine animals slaughtered underwent OFES and in the Netherlands the figure was 0.90 % of bovine animals slaughtered (*n* = 13,497) (1). In the Republic of Ireland between 2020 and September of 2021 0.02% (*n* = 662) of cattle were certified as OFES (8). However, there is a degree of under reporting of cattle processed as OFES in the official data (8).

The individual circumstances that affect whether injured cattle are fit enough to be transported to abattoirs for CS or whether other options, such as treatment, OFES or euthanasia, are preferable to reduce the risk of suffering has been reported in Ireland (6) and other parts of the EU (9), North America (10), and other parts of the world (2). The European Commission, Directorate General for Health and Food Safety, carried out audits in a number of EU member states where the main objective was to evaluate the operation of official controls and the enforcement of the applicable EU requirements. They included certain aspects of animal welfare and especially the evaluation of fitness for transport and slaughter. In the Netherlands the audit team were shown examples of fines, starting at 1,500 euro, which were imposed for the transport of unfit animals to abattoirs (11). In Spain auditors reported evidence of OVs identifying cows in the lairage considered as unfit to travel but had been transported to the abattoir contrary to the regulations (12).

In Canada it has been reported that the transportation of unfit cattle is a frequent cause of non-compliance with the *Health of Animals Regulations* (13, 14). The EU and a number of provinces in Canada have regulations and procedures in place for the OFES of cattle (15). In British Columbia OFES is mainly used for dairy cows and the types of injuries and conditions that led to OFES were similar to those reported for general dairy cow mortality on farms; these commonly include accidents and calving-related injuries (16). While Canada, the USA and Mexico each have codes and regulations governing the transport of farmed animals, there are shortcomings in their scope and enforcement which present significant challenges for ensuring animal welfare in each of these countries. This is particularly true in relation to transport of animals by road destined for slaughter (17).

A study published in March 2022 by the European Commission found that similar issues occur in the EU in relation to the transport of unfit end-of-career dairy cows, however, the research was unable to identify the scale of the problem (18). It identified economic factors as a major driver, for example that it is more expensive to slaughter unfit cows on farm rather than at an abattoir. There is a financial gain for farmers to opt for CS at abattoirs. A lack of understanding and varying interpretations of stakeholders' perceptions of what constitutes unfit also contributes to unsuitable cows being transported to abattoirs (18).

Most of the limited research about how industry professionals perceive OFES, has focused on veterinarian views about the process and challenges about managing it. In one study, Irish stakeholders reported a conflict between a veterinarian's professional duty to protect animal welfare and their client's desire to salvage the financial value of cattle through OFES (19). In another study, 89% of OVs working in bovine abattoirs in Ireland did not want to accept OFES carcasses, citing concerns about food safety risks and decreased meat quality (1).

Whereas anecdotal evidence has suggested that OFES is controversial, little is known about how the procedure is perceived by individuals involved. The aim of the research was to explore the experience and the perspective of OVs and PVPs regarding the management of acutely injured cattle. In this study we adopted a framework reported by Coleman and Hemsworth (20) to investigate the influence of capacity, willingness and opportunity on PVPs and OVs to operate OFES. Capacity includes variables such as technical skills, knowledge of animal care as well as ability to carry out tasks. Willingness refers to motivation, job satisfaction, attitude to the animals and work attitude. Opportunity considers working conditions, actions of co-workers and organizational policies and rules (20). The authors defined an acute injury as an injury that is severe, causes acute pain (21), has a sudden onset, is usually associated with a traumatic event and is more commonly locomotory. The most common severe traumatic events are fractures in particular fractures of the metacarpus and metatarsus, followed by the tibia, radius and ulna, humerus, and femur (22). This is distinct from non-acute injuries such as joint trauma or hoof cracks.

## Materials and methods

Two online surveys of PVPs and OVs working in Ireland, were conducted over a 7-week period between April and June of 2021, using Qualtrics^XM^.

### Materials

#### Design

Survey design followed a process of initial design, refinement, piloting and further refinement. Quantitative and qualitative questions were developed relevant to capacity, willingness and opportunity, in the context of the management of acutely injured cattle and the provision of OFES in Ireland. Quantitative questions included the demographic profile of the participants such as age, number of abattoirs regulated by OVs and number of PVPs in a practice. Qualitative questions were designed to investigate where information in relation to OFES is obtained and whether veterinarians have a policy in relation to OFES. The qualitative questions also explored level of agreement with statements using an 11-point Likert scale (0 = completely disagree; 10 = completely agree), and ranking questions (ranking of items on importance).

Both open-ended and closed questions were used in the surveys. Open-ended questions were used to assess OVs and PVPs attitudes, such as what they consider to be the positive and negative aspects of OFES, in relation to animal welfare as a procedure for dealing with acutely injured cattle. Closed questions involved offering respondents a number of defined response choices and were used also in questions such as what informs their knowledge about the management of acutely injured cattle. Regarding the number of acutely injured cattle, PVPs and OVs were asked to provide data for 2020. Separate surveys were designed for OVs and PVPs to take into account differences in their professional roles and responsibilities. A number of questions were common to both surveys, so that a comparison of both PVPs and OVs responses could be assessed.

The questionnaires for OVs and PVPs were divided into four parts: demographics and their place of employment (e.g., types of abattoirs or private practices); Capacity (e.g., knowledge and skills); Willingness (e.g., motivation); Opportunities (e.g., policy environment).

Surveys were designed followed by online piloting. Four PVPs in food animal practice and four OVs (two in the Local Authority and two in DAFM) were asked to complete the survey and fill in a feedback checklist. The two questionnaires are included in the Supplementary material.

### Methods

#### Recruitment

The PVP survey was distributed by Veterinary Ireland, the national representative group for veterinarians in Ireland, to members of their Food Animal Interest Group, using their database VetALERT (*n* = 673). In addition, a short article about the survey with a QR code was published in the May edition of The Veterinary Ireland Journal, to increase awareness and garner engagement. The OV survey was sent to all OVs (*n* = 38) working in Local Authorities and those working in Veterinary Public Health in DAFM (*n* = 72) *via* Email. Two reminders about the surveys were sent by email on 7th May and 17th May 2021. All data were anonymized.

#### Data analysis

Online survey data were automatically uploaded into Microsoft Excel from Qualtrics. The data were cleaned, and QualtricsXM a codebook of responses created. Each response was given a variable name and a numerical code.

For open-ended questions, responses were scanned for common themes by using thematic analysis (23). These common themes were also numerically coded.

Data were uploaded into IBM SPSS v27.0 followed by statistical analysis. Before data analysis could begin variables were defined.

The relationship between respondent demographics of age, gender, years qualified, location of qualification, and post graduate qualifications was investigated using linear logistic regression. Beta (β) the standardized coefficients were also calculated. These measure the strength of the effect of each individual independent variable to the dependent variable, ranging from 0 to 1 or 0 to −1 depending on the direction of the relationship. The closer the value is to 1 or −1, the stronger the relationship. The relationship between respondent's knowledge about the procedure of OFES and age, gender, years qualified, location of qualification, and post graduate qualifications was investigated using Spearman's rank order correlation. Spearman's rank order correlation coefficient are measures of the strength of linear associations between two variables. Spearman's rho correlation, *r*_*s*_, can take a range of values from +1 to −1 (24, 25). Explanatory variables were explored using Cramer's V (Negligible: V < 0.1, Weak: 0.1 ≤ V < 0.3, Moderate: 0.3 ≤ V < 0.5, Strong: V ≥ 0.5) (25).

Descriptive statistics were used to give summary statistics such as the mean (*M*), median (*Me*), standard deviation (*SD*) and interquartile range (*IQR*). Descriptive statistics were also used to analyze Likert scale questions. An 11-point Likert scale question was used to determine survey participants knowledge, opinion and experience of OFES, where 0 indicated zero knowledge, very low opinion or very bad experience and 10, indicated extensive knowledge, very good opinion or very good experience of the procedure.

Ordinal logistic regression was performed after dependent (outcome) and independent (predictor) variables were chosen for each of the factors that could influence decisions made by OVs and PVPs in relation to the management of acutely injured cattle and the use of OFES. Preliminary analysis was performed before ordinal logistic regression was done to assess whether the level of knowledge (dependent variable) displayed by PVPs and OVs was influenced by the medium (independent variables) they used to obtain knowledge. It was also used to analyze if the matters discussed with OVs, and PVPs (independent variable) influenced OVs level of knowledge (dependent variable). Ordinal logistic regression analysis again was used to assess if OVs and PVPs opinion or experience of OFES (dependent variables), was influenced by what they considered to be the positive and negative aspects of OFES (independent variable). The unstandardized coefficient estimates B (the expected change in log odds), which describes the relationship between an independent variable and a response was also calculated using ordinal logistic regression. A positive coefficient indicates that as the value of the independent variable increases, the mean of the dependent variable also increases and vice versa. Wald tests (Wald χ^2^) were used to determine if certain independent variables were significant. A *p* < 0.05 was deemed statistically significant for the purpose of the study.

## Results

In total 43 OVs, and 85 PVPs participated in the study and no participants were excluded. The response rate to the survey for PVPs and OVs was 12.6 and 39.0%, respectively.

Table 1 illustrates participant demographics. Logistic regression found a negative correlation between age and gender for OVs indicating female respondents were younger (β = −*0.5, p* ≤ 0.001; B = −11.8). In relation to PVPs, female respondents were also younger (β = −0.3, *p* ≤ 0.001; B = −9.1). The majority of PVPs (90.8%, *n* = 69) worked in food animal practice, and the median number of PVPs in these practices was four. Cattle and or sheep were treated by 93.5% (*n* = 58) of PVPs, whilst 67.9% (*n* = 55) of PVPs were practice partners and 32.1% (*n* = 26) were assistants. In terms of practices 89.3% (*n* = 75) operated in the Republic of Ireland, 7.1% (*n* = 6) in either the Republic of Ireland and Northern Ireland, or solely in Northern Ireland (3.6%, *n* = 3).

**Table 1:** Profile of Official Veterinarians and Private Veterinary Practitioners participants in the study.

**Variable**	**Categories**	**Official veterinarians**			**Private veterinary practitioners**		
		***n***	**Mean (SD)**	**%**	***n***	**Mean (SD)**	**%**
Age		41	53.8 (9.8)		83	47.9 (12.4)	
Gender	Male			72.1			74.1
	Female			27.9			24.7
	Other						1.2
Years qualified		41	29.4 (11.1)		80	24.1 (12.4)	
Country of qualification	ROI			90.3			79.8
	UK			2.4			7.1
	Other			7.3			13.1
Number of years an OV		39	15.5 (10.9)				
Number of years a PVP		39	13.2 (6.4)		80	24.1 (12.5)	
Type of practice	Food animal			78.9			90.8
	Companion			7.9			7.9
	Other			13.2			1.3
Area of expertise	Cattle/Sheep			55.2			93.5
	Companion						
	Other			44.8			6.5
Jurisdictions worked in	ROI			55.0			63.1
	ROI and UK			32.5			25.0
	ROI and other			12.5			11.9

The participant demographics indicated that OVs had a higher mean age than PVPs (53.8 ± 9.82 and 47.9 ± 12.49 years, respectively). The mean age of male OVs was higher than that of female OVs (57.3 ± 8.39, 45.5 ± 7.99 years, respectively). The majority of OVs were male 72.1% (*n* = 31) and the same gender balance was recorded for PVPs (males: 74.1%, *n* = 63 vs. females: 24.7%, *n* = 21), one PVP respondent selected other. OVs were longer qualified than PVPs (29.4 ± 11.15 and 24.18 ± 12.47 years, respectively). 53.7% (*n* = 22) of OVs and 32.5% (*n* = 26) of PVPs were qualified over 30 years. In relation to OVs, 78.9% (*n* = 29) had previously worked in food animal practice for on average 13.2 years (*n* = 39) and subsequently as OVs for on average 15.5 years (*n* = 39) (Table 1). A total of 68.3% (*n* = 56) of PVPs worked as a temporary veterinary inspector in an abattoir.

### Capacity

There was no statistically significant difference between level of knowledge of OVs (*Me* = 6, *IQR* = 4) and PVPs (*Me* = 6, *IQR* = 4) in relation to the management of acutely injured cattle using OFES. There was a moderate, negative correlation between knowledge and gender for OVs (*r*_*s*_ = −0.325, *n* = 29, *p* = 0.086), indicating that female OVs perceive that they are less knowledgeable about OFES for the management of acutely injured cattle. There was a negative relationship between OVs knowledge of OFES by using professional organizations to obtain this knowledge (B = −3.758, *p* = 0.007). In relation to PVPs, regulations contributed little to their knowledge about OFES (B = −1.087, *p* = 0.013).

Table 2 shows that regulations and guidelines were the most common sources of knowledge for both cohorts. However, PVPs were more inclined to use professional organizations than OVs (15.2%, *n* = 28; 5.1%, *n* = 4, respectively). Results indicate that 21.2% of PVPs (*n* = 39) obtained knowledge about the management of acutely injured cattle from other PVPs, and 21.8% of OVs (*n* = 17) stated that they only obtain knowledge from other OVs. A total of 32.4% of PVPs (*n* = 39) reported that they consulted with their peers and 27.0% of OVs (*n* = 17) stated they only consulted with their peers about certifying cattle for OFES.

**Table 2:** Factors influencing the Capacity/Knowledge of Official Veterinarians and Private Veterinary Practitioners regarding the management of acutely injured cattle in Ireland.

		**Official veterinarians**			**Private veterinary practitioners**		
**Variable**	**Categories**	***n***	**Median (IQR)**	**%**	***n***	**Median (IQR)**	**%**
Knowledge of OFES		29	6 (4)		52	6 (4)	
Post Grad Qualifications	Cert VPH			33.3			11.6
	Diploma/MSc/VPH			30.0			
	Cert companion						11.6
	Animal med						48.9
	Cert dairy herd						27.9
	Health						
	Other			36.7			
Obtain knowledge about the management of injured cattle	Regulations			33.4			29.6
	Guidelines			32.1			31.3
	OVs			21.8			0.5
	Pro organizations			5.1			15.2
	PVPs			3.8			21.2
	Other			3.8			2.2
Consult with the following when certifying/accepting cattle for OFES	PVPs			32.4			27.0
	OVs			0.0			27.0
	Abattoir owners			35.3			27.0
	Don't consult			17.6			10.8
	Other			14.7			8.2
Matters discussed by OVs with OVs on management of injured cattle	Enforcement of transport Regs			12.5			
	Enforcement of Regs			37.4			
	Lack of availability			18.8			
	Potential abuse of system			18.8			
	Other			12.5			
Matters discussed by OVs with PVPs on management of injured cattle	Consultation re OFES			22.8			
	Certification			50.0			
	Management of injured cattle			13.6			
	Other			13.6			
Matters discussed by PVPs with OVs on management of injured cattle	OFES						16.3
	Certification						36.7
	No discussion						38.8
	Other						8.2
Matters discussed by PVPs with PVPs on management of injured cattle	Enforcement of transport Reg						32.0
	Enforcement of Regs						36.0
	Lack of availability						14.0
	Abuse of system						4.0
	Other						14.0

Table 2 illustrates that the main topics discussed amongst OVs were enforcement of regulation, potential abuse of the system and lack of availability of OFES. OVs perceived that PVPs were lacking knowledge in relation to managing acutely injured cattle (B = −2.6, *p* = 0.032). The main topics discussed between OVs and PVPs were obligations in relation to certification and consultation prior to the slaughter of OFES cattle. The majority of PVPs stated that they do not have a consultation with OVs prior to OFES (38.8%, *n* = 19). The main matter discussed with other PVPs was the enforcement of regulations. Lower levels of knowledge of OFES by PVPs was significantly influenced by the lack of availability (B = −2.3, *p* = 0.018), and their knowledge of certification of OFES cattle (B = −2.6, *p* = 0.014).

### Willingness

Survey participants were asked about the issues that influence their decision making in relation to the management of acutely injured cattle (Table 3). OVs stated the views of the competent authority were their main influencer (37.0%, *n* = 20), while 19.4% (*n* = 33) of PVPs stated practice policy regarding managing acutely injured cattle was the main influencer. However, the positive and negative aspects of OFES, were not statistically significant in influencing OVs opinion about OFES. In relation to PVPs, positive aspects of OFES did not influence opinion, but the negative aspects were significantly influential for all variables except for food safety and other issues (Table 3). PVPs have a negative opinion in relation to how the system of OFES may be abused (B _=_ −2.9).

**Table 3:** Factors influencing the willingness of Official Veterinarians and Private Veterinary Practitioner regarding the use of on-farm emergency slaughter for the management of acutely injured cattle in Ireland.

**Variable**	**Categories**	**Official veterinarians**			**Private veterinary practitioners**		
		***n***	**Median (IQR)**	**%**	***n***	**Median (IQR)**	**%**
Influences OVs decision in relation to managing acutely injured cattle	Competent authority			37.6			
	OVs			15.1			
	PVPs			9.5			
	Professional Organizations			7.6			
	Abattoir owners			17.0			
	Other			13.2			
Influences PVPs decision in relation to managing acutely injured cattle	Practice policy						19.4
	Fellow PVPs						15.9
	Farmers						16.5
	Abattoir owners						14.1
	Professional organizations						9.3
	OVs						12.4
	Other						12.4
Positive aspects of OFES	Prompt relief of welfare			38.5			24.9
	No transport issue			18.5			19.1
	Other welfare issues			33.8			29.4
	Economics			1.5			18.5
	Nothing positive			1.5			0.6
	Other			6.2			7.5
Negative aspects of OFES	Lack of availability			18.3			22.4
	No prompt relief of welfare			25.0			22.4
	Other welfare issues			18.3			11.2
	System open to abuse			16.7			10.0
	Food safety issues			6.7			4.6
	Economics						9.4
	Other			15.0			20.0
Time frame for managing acutely injured cattle	< 12 h			48.3			56.0
	< 24 h			32.3			35.7
	Other			19.4			11.3
Experience of OFES		26	5 (5)		78	5 (6)	
Opinion of OFES		27	5 (3)		79	5 (4)	

Regarding OVs, results indicated that the positive aspects of OFES had no influence on their experience of OFES while negative aspects were significant for the prompt relief of animal welfare (B = 37.5, *p* = 0.039). OVs perceived that OFES did not provide for prompt relief of animal welfare. Among PVPs that used OFES, the relationships between their experience and the positive aspects of OFES in relation to welfare and economics were statistically significant (B = −1.9, *p* = 0.043; B = −2.8, *p* = 0.008, respectively). Regarding experience and negative aspects of OFES, abuse of the system and negative economic outcomes were significant (B = −3.2, *p* = 0.044; B = −5.1, *p* = 0.004, respectively). Both positive and negative aspects of OFES influenced PVPs experience in a negative manner. Spearman's correlation was used to determine if there was a correlation between OVs and PVPs opinion and experience. OVs developed a more positive opinion of OFES when they had more experience of the procedure (*r*_*s*_ = 0.741, *n* = 25, *p* = < 0.001). There was no correlation between PVPs opinion and experience on the use of OFES for the management of acutely injured cattle.

There was a consistency amongst the cohorts in relation to the positive and negative aspects of OFES, more OVs considered that OFES provided a prompt relief of a welfare issue than those with a contrary view (38.5%, *n* = 25, 25%, *n* = 15, respectively). A similar proportion of PVPs agreed and disagreed that OFES provided a prompt relief of a welfare issue (24.9%, *n* = 43; 22.4%, *n* = 38, respectively) (Table 3).

The timeframe for managing acutely injured cattle was deemed by 48.4% (*n* = 15) of OVs and 56.0% (*n* = 47) of PVPs to be < 12 h, while 32.3% (*n* = 10) of OVs and 35.7% (*n* = 30) of PVPs indicated that < 24 h was an appropriate timeframe (Table 3).

### Opportunity

The mean number of abattoirs regulated by OVs (*n* = 39) was 4.2 (*SD* = 4.65), although 48.7% (*n* = 19) regulated one abattoir. Of these abattoirs, 61.5% (*n* = 24) accepted acutely injured cattle and 21.6% (*n* = 8) accepted OFES cattle. Two abattoirs accepted both acutely injured and OFES cattle. Participants reported a total of 343 OFES cases and 377 injured cattle were transported to abattoirs and processed as CS cattle in 2020.

A total of 63.3% (*n* = 27) of OVs and 44.4% (*n* = 36) of PVPs were not aware of any abattoirs providing for OFES within 100 km of their place of work. Where OFES is provided, 75% (*n* = 9) of OVs reported that abattoir personnel perform the operation. The majority of OVs (84.4%, *n* = 27) and PVPs (89.3%, *n* = 75) reported that they would like to see OFES being provided nationwide as a means of managing acutely injured cattle. Whilst 89.3% (*n* = 25) of the competent authorities (DAFM and LAVS) reported that they had a standard operating procedure in relation to OFES only 34.6% (*n* = 28) of PVP practices had a practice policy in relation to OFES and the majority of practice policies were in favor of its use (*Me* = 7, *IQR* = 3). The opinion on the rules/policies that enable OFES were similar for OVs and PVPs (*Me* = 5, *IQR* = 5) and (*Me* = 5, *IQR* = *2*), respectively (Table 4).

**Table 4:** Factors influencing the availability of on-farm emergency slaughter for the management of acutely injured cattle in Ireland from the perspectives of Official Veterinarians and Private Veterinary Practitioners.

		**Official veterinarians**			**Private veterinary practitioners**		
**Variable**	**Categories**	***n***	**Median (IQR)**	**%**	***n***	**Median (IQR)**	**%**
Is practice policy in favor of OFES					27	7 (3)	
Opinion on rules/policies on OFES		24	5 (5)		72	5 (2)	
Example of how OVs think the rules/policies are too restrictive	Not too restrictive			16.7			
	Restricts type of abattoir			25.0			
	Restricts type of animal			25.0			
	Other			33.3			
Example of how PVPs think the rules/policies are too restrictive	Not too restrictive						3.6
	Procedures/Guidelines too restrictive						25.0
	Other						71.4
Recommend the following changes	Subsidize the procedure			14.7			15.8
	More training			26.8			15.8
	Improve availability			26.8			39.1
	Enforce regulations			7.3			6.0
	Allow meat from OFES to be retailed			7.3			0.8
	Increase awareness						9.0
	Other			17.1			13.5
Outline how the potential of OFES could be realized	More availability			33.3			34.0
	More types of animals able to avail of OFES			10.0			
	Subsidize			23.3			15.5
	More information			20.0			30.1
	Allow sale on open market			6.7			3.9
	Other			6.7			16.5
Anything else you would like to add in relation to the management of acutely injured cattle	More availability			38.5			28.1
	Farmers unaware of transport regulations			15.4			12.7
	Animal welfare and food safety should be foremost			23.0			20.5
	Good oversight required			15.4			10.5
	Amend procedures			0.0			15.4
	Other			7.7			12.8

**Figure 1:**
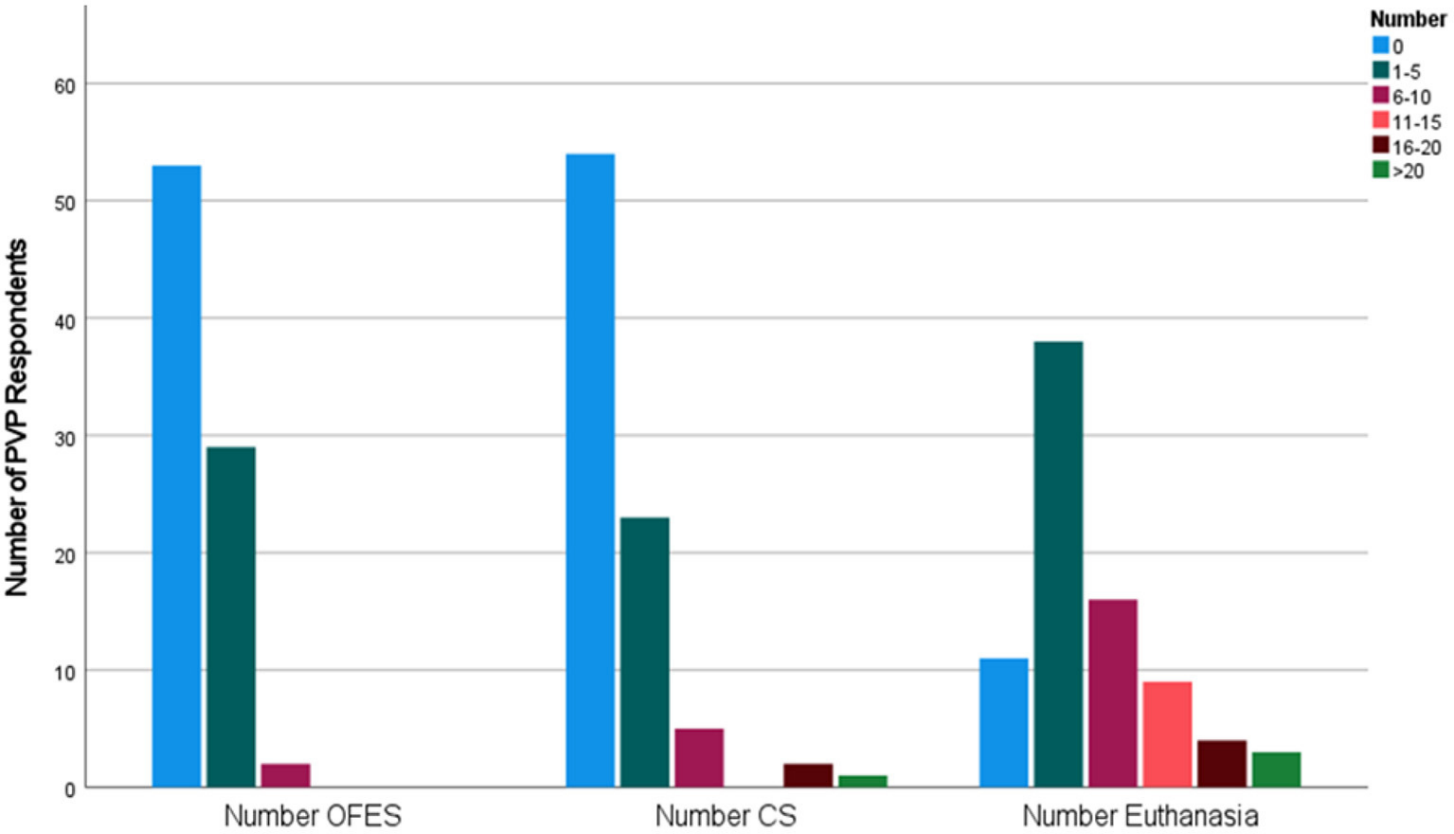
The number of acutely injured cattle certified for on-farm emergency (OFES) slaughter and casualty slaughter (CS) or euthanised reported by Private Veterinary Practitioners (*n* = 85) in 2020.

In relation to PVPs certifying acutely injured cattle, 62.4% (*n* = 53) did not certify cattle for OFES nor for CS in 2020. During this study period, 34.5% of PVPs (*n* = 29) certified from 1 to 5 cattle for OFES and a similar proportion (28.2%, *n* = 24) certified from 1 to 5 cattle for CS. In relation to euthanasia, 13.6% (*n* = 11) of PVPs did not perform euthanasia on any injured cattle, while 46.9% (*n* = 38) euthanized 1–5 and 19.8% (*n* = 16) euthanized 6–10 (Figure 1).

**Figure 2:**
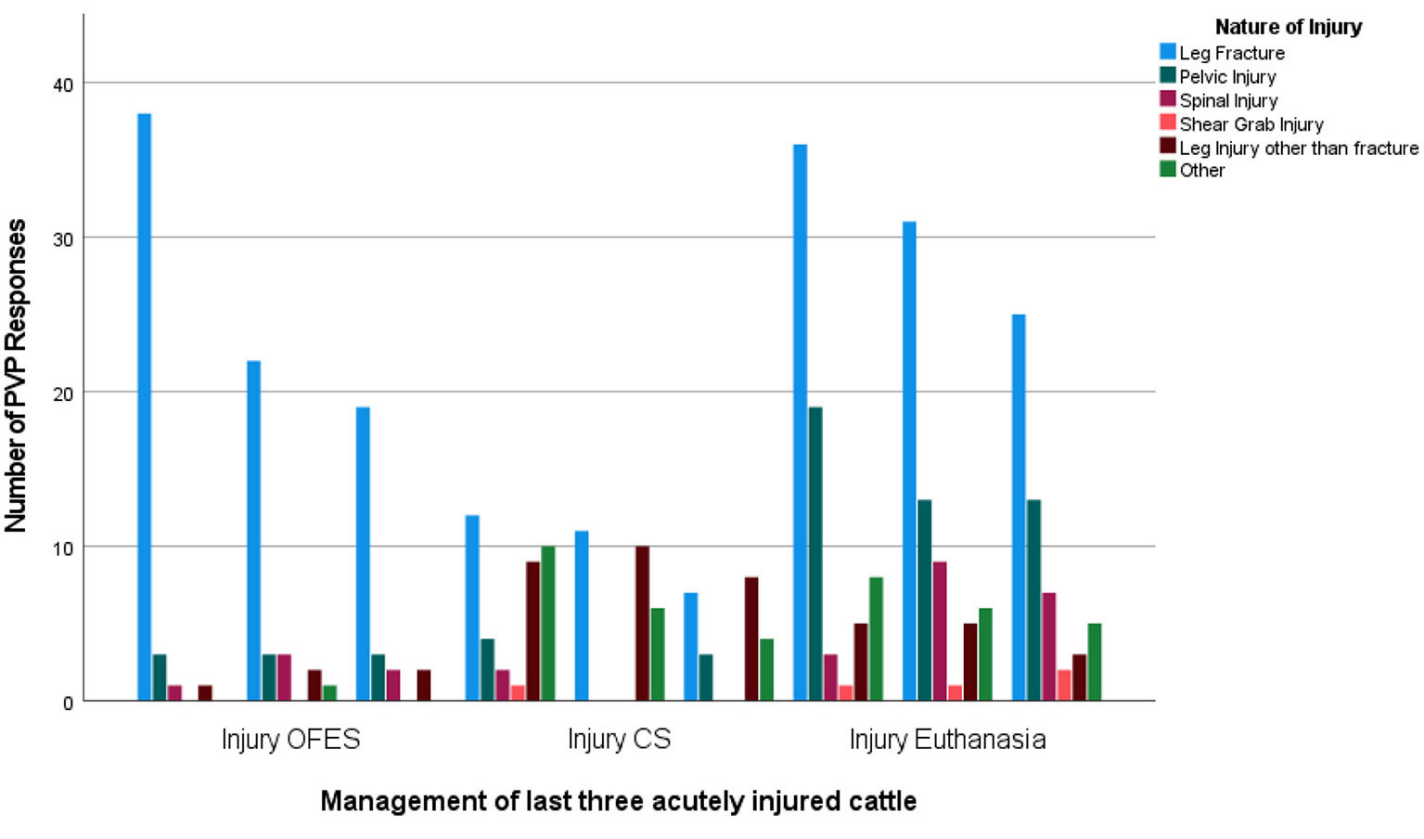
The type of acute injury for the last three cattle resulting in on-farm emergency slaughter (OFES), casualty slaughter (CS) and euthanasia as reported by Private Veterinary Practitioners (*n* = 72) in 2020.

Limb fracture was the most common cause of acute injury for OFES cattle reported by PVPs (79.0%, *n* = 79), compared to 34.5% (*n* = 30) of CS cattle and 47.9% (*n* = 92) of acutely injured cattle that had to be euthanized. PVPs reported pelvic injuries as the second most common reason for OFES (9.0%, *n* = 9) and euthanasia (23.4%, *n* = 45) (Figure 2).

**Figure 3:**
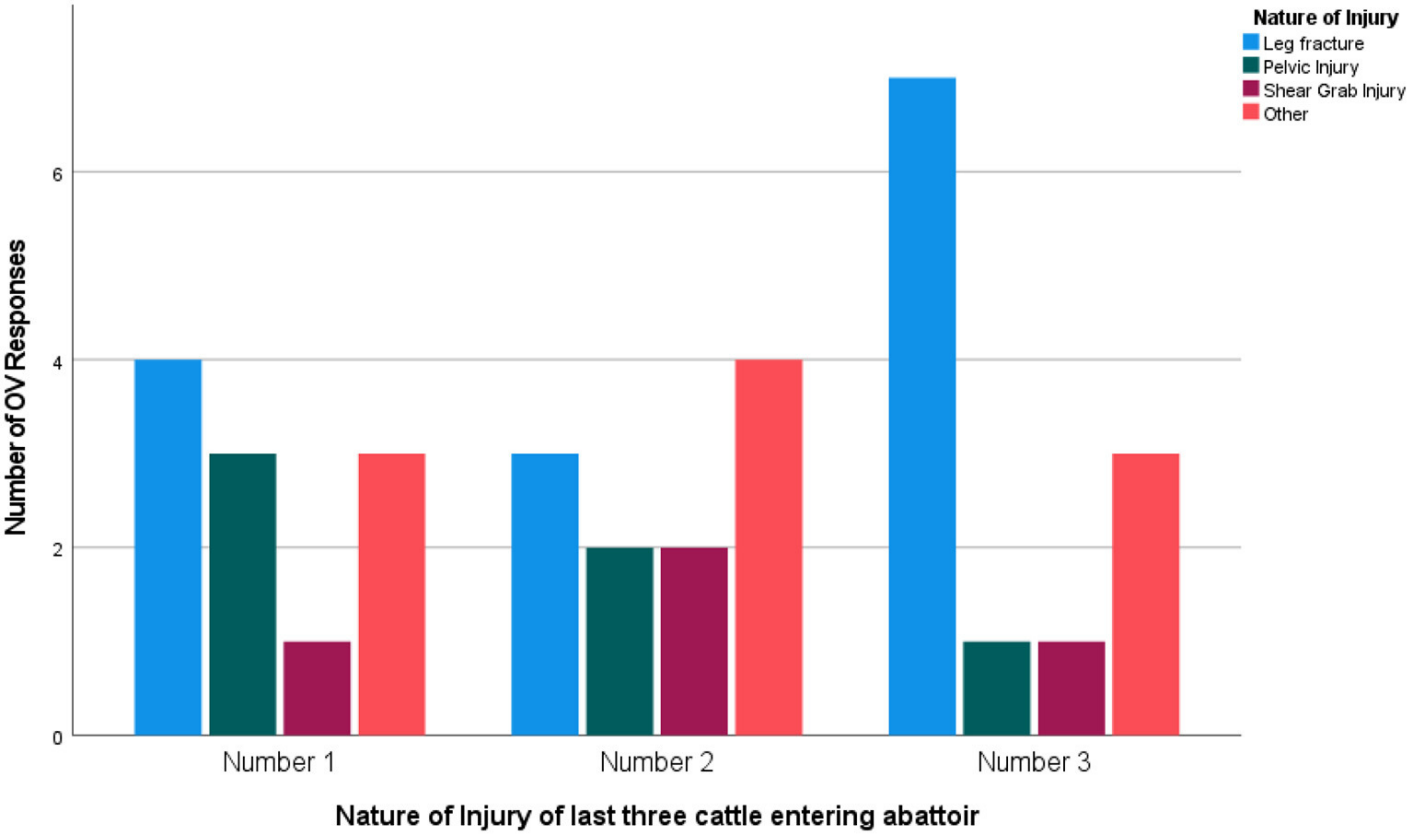
The type of acute injury reported by Official Veterinarians (*n* = 13) for the last three cattle certified for casualty slaughter in 2020.

According to OVs, locomotory disorders were the most common reason for OFES, with 86.7% (*n* = 13) citing a fracture of the limb as the reason for OFES and leg injuries other than fractures was the second reason (13.3%, *n* = 2). 41.2% (*n* = 14) of CS cattle had a leg fracture, 17.6% (*n* = 6) had a pelvic injury and 11.8% (*n* = 4) had an injury caused by machinery (e.g., a shear grab) (Figure 3).

A total of 66.7% of OVs (*n* = 16) and 78.1% of PVPs (*n* = 57) believed that OFES had potential. When asked what changes they would recommend to improve the procedure of OFES, 33.3% of OVs (*n* = 10) and 34.0% of PVPs (*n* = 35) reported improved availability. Further comments stated more information/education of stakeholders and subsidizing the procedure would be of benefit (Table 4). Further comments included that animal welfare and food safety should be foremost and that farmers are unaware of transport regulations.

## Discussion

The aims of this study were to evaluate the perceptions of veterinarians, working in Ireland, on the use of OFES for the management of acutely injured cattle and to evaluate the influence of capacity, willingness and opportunity on their ability to operate OFES. The Veterinary Council of Ireland reported in October 2020 that there were 3,058 registered veterinary practitioners in Ireland (26), of which 54.8% (*n* = 1,675) were male and 43.1% (*n* = 1,319) were female (26). In total 43 OVs and 85 PVPs participated in the study. The role of PVPs who work in food animal practice on the island of Ireland may extend to meat inspection if they are employed as temporary veterinary inspectors in abattoirs. In both surveys over 70% of respondents were male and on average qualified for more than 24 years and almost 70% were practice partners. The PVP survey was distributed to members of the Food Animal Interest Group of Veterinary Ireland, the national representative group for veterinarians in Ireland, and this may have contributed to survey participants demographics. However, practice partners create practice policy so their views are important and would have a major influence on how the practice manage acutely injured cattle. The Veterinary Council of Ireland's list of registrants reported that 12.4% of registered Veterinarians were born in the 1950s while 15.5% were born in the 1960s and 22% in the 1970s (27), which would indicate that a larger number of older individuals responded to the surveys. Until recent years, the veterinary profession has had a male gender bias, which is likely to explain the demographics of respondents particularly those who have been qualified since 1997 (28). A number of studies on veterinary attitudes toward aspects of animal welfare have reported gender and generational differences. For example, a survey on pain scoring showed higher pain scores reported by female than male veterinarians, while those graduating before 1990 recorded lower pain scores compared to those graduating after 2010. As the majority of respondents in the current study were male and had graduated pre-2010 (29), the results may reflect a gender and generational bias in relation to how acutely injured cattle are managed. The other factor that may have influenced survey results is the small sample size for PVPs, which has the potential to undermine the internal and external validity of a study (30). However, while the sample size for PVPs was small, not all PVPs work in food animal practice. Furthermore, not all PVPs working in food animal practice are members of Veterinary Ireland. In contrast, the OV survey was representative of Veterinarians working in Veterinary Public Health in Ireland.

Knowledge is a key characteristic underpinning attitude and behavior toward animal welfare (20, 31). There was no significant difference between the level of knowledge of OVs and PVPs. Both groups stated that regulations and guidelines provided their primary source of information, which is consistent with the requirements of the Veterinary Council of Ireland, Code of Professional Conduct, “*Veterinary practitioners should familiarize themselves with the requirements of legislation and standards that are relevant to their area of practice”* (32). There is a consistency amongst the two cohorts of veterinarians in that one of the main matters discussed amongst OVs and PVPs was enforcement of regulations. However, this finding is at odds with the large number of cattle being transported for CS, and that a number of these have fractured legs, would indicate that some veterinarians, though cognizant of the rules in relation to the transport of welfare compromised cattle, are willing to both certify these cattle for transport (PVPs) and accept them into abattoirs for slaughter (OVs).

The highest percentage for both the positive and negative aspects of OFES for each cohort of veterinarians were contradictory regarding whether OFES provides a prompt relief for animal welfare. This is consistent with other studies, such as farmers' perceptions of OFES in British Columbia: “*OFES was perceived to be positive for cow welfare because it decreases the amount of time that a cow may suffer in transport or during unsuccessful rehabilitation on the farm but in contrast, other participants believed that OFES is negative for cow welfare because it prolongs animal suffering”* (33). These participants perceived OFES in positive and negative ways based on individual values, perceptions about the legitimacy of OFES, and concern over social responsibleness (32). This may be similar to the survey data reported here but these factors were not explored. The correlation between OVs opinion and their experience of OFES may indicate that OVs who do not regulate abattoirs that provide for the OFES of cattle may have developed a negative perception of the procedure. It has been shown in other studies, that a positive opinion, and thus a more positive attitude toward OFES for managing acutely injured cattle could have a more positive effect on the procedure becoming more widely available (34).

The majority of OVs regulated one abattoir and there was a differential of 39.9% between those that accept CS and OFES cattle, thus indicating that there is more availability for farmers in Ireland to have cattle slaughtered using CS rather than by OFES. The concern in relation to animal welfare is that a number of CS cattle have a fracture of the leg and should not be transported. A study conducted in the Republic of Ireland between 2006 and 2008, demonstrated that, of cattle consigned under veterinary certification to four large abattoirs, over 60% of the cattle could have been designated for OFES, if the procedure was available (2, 35). Without examining the conditions that cattle certified for CS were suffering, it cannot be determined how many of these transported cattle were in contravention of the regulations pertaining to the transport of injured cattle (4). This issue of non-compliance is not unique to Ireland, however there are examples of good practice. The official audits carried out by the European Commission in a number of EU member stated that in Malta there is an efficient system in place for emergency slaughter at farms of animals unfit for transport. This has dramatically reduced the number of animals deemed unfit for transport arriving at abattoirs (36). Therefore, not alone does there need to be a change in relation to the use of CS in Ireland but also in other parts of Europe, and if OFES is provided it helps to reduce the number of cattle transported for CS.

In relation to how acutely injured cattle may be managed, OFES, CS and euthanasia were investigated. Treatment was not investigated because it was not considered to be a common viable option for acutely injured cattle, but it has been reported that this may happen in a small number of cases (37). The cost of treatment, except possibly in show cattle, and the level of care post-treatment makes the procedure difficult for farmers to manage and economically non-viable in most cases (38). The most common method of managing acutely injured cattle was by euthanasia (39). This indicates that the majority of PVPs are complying with the rules and regulations.

OVs and PVPs opinion of OFES was marginally positive, with the exception of the rules and policies that govern the procedure. For example, OVs considered that it was restrictive regarding the type of abattoirs and the type of animals that are deemed suitable. PVPs provided a spectrum of reasons, with the restrictive nature of the procedure being the most commonly cited. Restrictions on the type of abattoir permitted to perform OFES may be due to national agreements with third parties such as United States Department of Agriculture Approval to sell meat to the United States. Under Enact S. 1779 & H.R. 3931 the Downed Animal Protection Act prohibits inspectors at abattoirs from approving meat from non-ambulatory livestock for human consumption and requires their humane euthanasia (40). In relation to Local Authority abattoirs, refusal to permit OFES may be due to the abattoir owner's perception that the intake of OFES carcasses will have an adverse effect on their business, and under legislation they are not required to facilitate the process (1, 41).

The majority of PVPs and OVs stated that there was no abattoir within 100 km of their practice providing for OFES. This lack of availability of OFES is likely to increase the risk of acutely injured cattle being sent for CS and non-compliance with the regulations. Dairy and beef production represent the main livestock sectors in Ireland. In 2016 there were 137,500 farms in the Republic of Ireland of which ~81% farmed cattle, with a total cattle population of 6.9 million. Regional differences are evident characterized by 60% of the cattle farms located in the Southeast region. In contrast 38.0% of cattle farms were located in the Border, Midland, Western regions (42). Currently there are 12 abattoirs in the Republic of Ireland permitting OFES of cattle, of which 50% are in two of the 26 counties: four in County Cork (southeast) and two in County Mayo (west). Given the scale of cattle farming in Ireland, improved provision of OFES is warranted to support regulatory compliance and prevent unnecessary suffering of acutely injured animals. In 2016 there were 232,524 on farm deaths which represents 3.5% of the total cattle population (43).

In the majority of abattoirs that permit OFES, the on-farm procedure is performed by abattoir personnel, and not PVPs. The majority of veterinarians would like to see OFES being available nationwide, and whilst most veterinary practices of the PVPs surveyed do not have a policy on OFES, of those that do, the policy is in favor of the procedure. The lack of availability of OFES has the potential for a conflict of interest to arise for PVPs, where the farmer may request CS, which may infringe the regulations if the animal is unfit for transport. Such ethical challenges have been previously reported as a key issue by veterinary professionals (6, 19). The lack of availability of OFES is also an issue in other Member States of the EU. The provision of OFES can alleviate matters as reported by the European Commission which stated “*that in one region in Poland, the incidence of unfit animals transported fell by 60% between 2013 and 2014. During the same period the number of animal's emergency slaughtered outside the slaughterhouse in this region increased by 235%”* (44).

In terms of future opportunities for improving the availability of OFES, the two cohorts of veterinarians agreed that more training for stakeholders and subsidizing the procedure could help in making OFES more accessible to all stakeholders. While subsidizing the process may be difficult to operate, the funding of small abattoirs and especially those that provide the service may be beneficial (45). When OVs and PVPs were asked if they had any further comments, in addition to the above, participants noted that food safety and animal welfare should be foremost and that farmers are unaware of the regulations. Food safety should not be an issue if the guidelines in relation to OFES are followed and suggests that further training is required to address concerns related to the procedure (34, 46).

The number of acutely injured cattle that undergo OFES in Ireland is low by international standards (1, 47). The results of this survey indicate that the CS of acutely injured cattle is still taking place and that a cohort of PVPs certify injured cattle for transport and a number of OVs accept them into abattoirs for slaughter. Persistence of animal welfare issues in other contexts has been attributed to accepted norms (16), underestimation of the problem (32), or poor perception of the mitigation strategies (32), which in this case would correspond to negative views of OFES. While acknowledging that it is challenging for veterinarians when presented with acutely injured cattle, especially in cases with limb fractures where a farmer may request CS, veterinarians should prioritize animal welfare and their ethical and professional responsibilities to their practice, other clients, other veterinary professionals, the EU transport regulations and other key stakeholders in animal agriculture. PVPs and their clients need to develop a mutual trust when dealing with these cases and take the best animal welfare option for the acutely injured animal (48, 49). Survey responses stating that farmers are unaware of the regulations suggests that acutely injured cattle have been transported by farmers or hauliers for CS without certification.

Cullinane et al. (5) stated that the first step in changing the management practices of OFES/CS animals is to improve stakeholders' awareness of their legal and moral obligations toward the welfare of animals concerned (5). The use of social psychology methodology and frameworks, such as the theory of planned behavior, can provide a detailed insight into human attitudes and behaviors that affect animal welfare. This approach can reveal the most important specific factors to consider when training and educating personnel who have direct responsibility for the humane treatment of animals (50). The RESET Mindset Model, used to change Dutch veterinarians' attitudes to the use of antibiotics in dairy cattle in the Netherlands, is an example of this change behavior model. This model contains the most important cues to change human behavior, namely regulations, education, social pressure and economics. To change behavior of groups in order to reach a tipping point, it is of utmost importance to use all of the cues (51). The mindset and perceptions that still prevails among some veterinarians in certifying and allowing cattle to be transported for CS will have to be addressed, along with methods to make OFES more available so as to provide veterinarians and farmers with an alternative to CS and euthanasia (52).

The limitations in relation to OFES are mainly due to lack of availability, with the majority of DAFM abattoirs not operating the procedure due to international trade agreements, and the majority of Local Authority not providing the service due to abattoir owners perception that it would have a negative influence on their business. The best direction for OFES to become more available for the management of acutely injured cattle is for more Local Authority abattoirs to provide for the service. The provision of subsidies for the procedure could help in expanding the service. It was reported that a number of injured cattle that undergo OFES are not recorded on the DAFM Animal Identification and Movement database, however, the figures were not collated (8), and the number of injured cattle that undergo CS or are euthanized are not available. Data capture is essential to an evidence-based approach, to inform how acutely injured cattle are managed and determine the requirement for OFES in Ireland.

## Conclusion

Results from both surveys indicate that OVs and PVPs views on issues concerning the management of acutely injured cattle are similar. The survey results reported here indicate that PVPs and OVs are positive toward the use of OFES, are aware of their legal and moral obligations in relation to animal welfare but that a lack of availability of OFES due to a number of factors such as FBO perceptions of the procedure and financial viability, have contributed to it not being used extensively for managing acutely injured cattle. Euthanasia is the most common method of managing acutely injured cattle by PVPs which demonstrates compliance with regulations on fitness to transport, in the absence of OFES. Improving the availability of OFES and providing training to stakeholders about the procedure would help to reduce the number of acutely injured cattle that go for CS, euthanasia or possibly treatment.

## Data availability statement

The raw data supporting the conclusions of this article will be made available by the authors, without undue reservation.

## Ethics statement

The studies involving human participants were reviewed and approved by UCD Human Research Ethics Committee. The patients/participants provided their written informed consent to participate in this study.

## Author contributions

PM, AH, AM, and FS conceived the study and designed the survey collaborated in the data collection. PM analyzed the data and drafted the manuscript. AH supervised the study and scientific work. AH, AM, and FS reviewed the manuscript. All authors read and approved the final manuscript.

## Funding

The study was funded by a grant from the Department of Agriculture, Food and the Marine, Ireland (Grant No. R20575).

## Conflict of interest

The authors declare that the research was conducted in the absence of any commercial or financial relationships that could be construed as a potential conflict of interest.

## Publisher's note

All claims expressed in this article are solely those of the authors and do not necessarily represent those of their affiliated organizations, or those of the publisher, the editors and the reviewers. Any product that may be evaluated in this article, or claim that may be made by its manufacturer, is not guaranteed or endorsed by the publisher.

## Supplementary material

Supplementary Material OV and the PVP survey.

## Abbreviations

CS: casualty slaughter
DAFM, Department of Agriculture: Food and the Marine
EU: European Union
IQR: interquartile range
LAVS: local authority veterinary service
M: mean
Me: median
OFES: on farm emergency slaughter
OR: odds ratio
OV: Official Veterinarian
PVP: Private Veterinary Practitioners
SD: standard deviation.
