# Supplementary material for: Management of acutely injured cattle by on farm emergency slaughter: Survey of veterinarian views
Source: Front Vet Sci. 2022 Nov 10;9:976595. doi: 10.3389/fvets.2022.976595 (PMC9686391; doi:10.3389/fvets.2022.976595)
Supplement: Supplementary Material — OV and the PVP survey. [file Table_1.DOCX]

Survey on the Management of Acutely Injured Cattle/OVs

Q1                                                                                                                                                                                                                          
 ***A questi****onnaire-based survey on the management of acutely injured cattle in the Republic of Ireland.*

*Dear Colleague,* I *am a Veterinarian working in Veterinary Public Health as a Veterinary Officer with Mayo County Council.  I am conducting a project with Associate Professor Alison Hanlon, UCD School of Veterinary Medicine, Assistant Professor Flavia Santos, UCD School of Psychology and Dr. Aideen McKevitt, UCD School of Agriculture and Food Science. The survey should be answered by Official Veterinarians working in Veterinary Public Health*   *The survey focuses on the management of acutely injured cattle in the Republic of Ireland. An acute injury is an injury that is severe, causes acute pain, has a sudden onset, is usually associated with a traumatic event and is commonly locomotory. The overall aim of the project is to determine how acutely injured cattle are managed in Ireland. The survey considers three areas:1. The methods used by OVs' to manage acutely injured cattle.* *2. The OVs' opinions and experience in relation to on farm emergency slaughter.* *3. The OVs' opinions on the rules and policies in relation to on farm emergency slaughter.*

 *All data will be anonymised and stored securely in compliance with UCD rules and regulations.* *The survey will take approximately 10 - 12 minutes and you must consent to participate in the survey.*   *The survey closes on 21st May.* *Thanking you,* *Yours sincerely,* *Paul McDermott, MVB, MSc (VPH), MRCVS.* *paul.mc-dermott.1@ucdconnect.ie*

- Yes, I Consent
- No, I do not consent

Q2 What is your age?

________________________________________________________________

Q3 What is your gender?

- Male
- Female
- Other

Q4 How many years have you been qualified as a Veterinarian?

________________________________________________________________

|  |
| --- |

Q5 Where did you qualify?

- Republic of Ireland
- UK
- Other (please specify) ________________________________________________

Q6 The post-graduate qualifications that I have undertaken are (tick all that apply)

- Graduate Cert Small Animal Medicine
- Graduate Cert Dairy Health
- Graduate Cert Equine Sports Medicine
- Graduate Cert Canine Sports Medicine
- Cert Veterinary Public Health (VPH)
- Diploma VPH
- MSc VPH
- MVM
- Other (please specify) ________________________________________________

Q7 **How many** **years** have you been an Official Veterinarian (OV) in Veterinary Public Health (numeric value)

________________________________________________________________

Q8 **How many** **years** were you a Private Veterinary Practitioner (PVP) (numeric value)

________________________________________________________________

Q9 My main area of **expertise** as a PVP was (select only one)

- Food Animal (please specify species)
- Companion Animal (2)
- Equine (3)
- Other (please specify) (4)

________________________________________________

Q10 The **jurisdictions** I have worked in are (tick all that apply)

- Republic of Ireland (1)
- UK (2)
- Other, please specify (3) ________________________________________________

Q11 The **number** of slaughterhouses I regulate is (numeric only)

________________________________________________________________

Q12 The **number** of slaughterhouses I regulate that accept acutely injured cattle is (numeric only)

________________________________________________________________

Q13 The **number** of slaughterhouses I regulate that provide the On Farm Emergency Slaughter **(OFES)**service to farmers is (numeric only)

________________________________________________________________

Q14 **The number** **of OFES** cattle that have been processed in the premises I regulate between the 1 Jan to 31 Dec 2020 is (numeric only)

________________________________________________________________

Q15 **The nature of the injury** of the last three cattle that **OFES** was performed on was

- 1 ________________________________________________
- 2 ________________________________________________
- 3 ________________________________________________

Q16 **The number** **of casualty slaughtered cattle** that have been processed in the premises I regulate between the 1 Jan to 31 Dec 2020 is

________________________________________________________________

Q17 The nature of the injury of the last three cattle that **casualty slaughter** was performed on was

- 1 ________________________________________________
- 2 ________________________________________________
- 3 ________________________________________________

Q18 **The number of abattoirs** I am aware of within a 100 Km radius of my workplace that provide the service of **OFES** is (numeric value)

________________________________________________________________

Q19 In the slaughterhouse I regulate the **OFES procedure** is performed by the

- Abattoir Personnel (1)
- PVP (2)

Q20 **My knowledge about**the procedures regarding **OFES** is, where **0 is my knowledge is extremely limited and 10 is I am very knowledgeable**

|  | 0 | 1 | 2 | 3 | 4 | 5 | 6 | 7 | 8 | 9 | 10 |
| --- | --- | --- | --- | --- | --- | --- | --- | --- | --- | --- | --- |

| Number () | 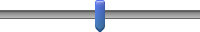 |
| --- | --- |

Q21 My **knowledge** about the management of acutely injured cattle is informed by (tick all that apply)

- Guidelines
- Regulations
- Other OVs
- PVPs
- Professional veterinary organisation
- Others (specify) ________________________________________________

Q22 **T**he **time frame** for managing cattle with an acute injury should not exceed

- 12hrs
- 24hrs
- 48hrs
- Other (specify) ________________________________________________

Q23 I **consult** with the following about accepting **OFES** cattle (tick all that apply)

- PVP
- Abattoir owners
- I don't consult
- Other (specify) ________________________________________________

Q24 **My experience** about the procedure of **OFES** on a scale of 0-10 is,
 where **0 is extremely bad** and **10 is extremely good**

|  | 0 | 1 | 2 | 3 | 4 | 5 | 6 | 7 | 8 | 9 | 10 |
| --- | --- | --- | --- | --- | --- | --- | --- | --- | --- | --- | --- |

| Number () | 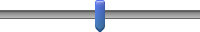 |
| --- | --- |

Q25 **My** **opinion** about the procedure of **OFES** on a scale of 0-10 is,
 where **0 is extremely negative** and **10 is extremely positive**

|  | 0 | 1 | 2 | 3 | 4 | 5 | 6 | 7 | 8 | 9 | 10 |
| --- | --- | --- | --- | --- | --- | --- | --- | --- | --- | --- | --- |

| Number () | 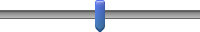 |
| --- | --- |

Q26 I **would like to see OFES** as a method for dealing with acutely injured cattle **available nationwide**

- Yes
- No

Q27 Is a **butcher shop** associated with the slaughterhouse?

- Yes
- No

Q28 The three **positive aspects of OFES**, in relation to **animal welfare**, when dealing with acutely injured cattle are

- 1 ________________________________________________
- 2 ________________________________________________
- 3 ________________________________________________

Q29 The three **negative aspects of OFES,** in relation to **animal welfare**, when dealing with acutely injured cattle are

- 1 ________________________________________________
- 2 ________________________________________________
- 3 ________________________________________________

Q30 I would recommend the following **three** **changes** to the current **OFES** procedure

- 1 ________________________________________________
- 2 ________________________________________________
- 3 ________________________________________________

Q31 What matters have you **discussed with other OVs** about the management of acutely injured cattle?

________________________________________________________________

________________________________________________________________

________________________________________________________________

________________________________________________________________

________________________________________________________________

Q32 What matters have you **discussed with PVPs** on the management of acutely injured cattle?

________________________________________________________________

________________________________________________________________

________________________________________________________________

________________________________________________________________

________________________________________________________________

Q33 I work in a Competent Authority that has **a Standard Operating Policy** in relation to **OFES**

- Yes
- No

Q34 In your opinion are the **policy/rules** in the **Standard Operating Policy** that enables the procedure of OFES are on a scale of 0-10,
 **where 0 is very restrictive and 10 is not restrictive**

|  | 0 | 1 | 2 | 3 | 4 | 5 | 6 | 7 | 8 | 9 | 10 |
| --- | --- | --- | --- | --- | --- | --- | --- | --- | --- | --- | --- |

| Number () | 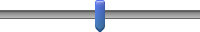 |
| --- | --- |

Q35 If the **policy/rules** are too restrictive please give three examples

- 1 ________________________________________________
- 2 ________________________________________________
- 3 ________________________________________________

Q36 My **decision making** in relation to the management of acutely injured cattle is influenced by (tick all that apply)

- Competent Authority Policy
- Other OVs
- PVPs
- Abattoir owners
- Veterinary Organisations
- Other (please specify) ________________________________________________

Q37 Does OFES have any **unrealised potential?**

- Yes
- No
- I don't know

Q38 If yes, can you outline three ways **this potential** may be realised so as to encourage wider adoption of the procedure

- 1 ________________________________________________
- 2 ________________________________________________
- 3 ________________________________________________

Q39 Is there **anything else** you would like to add in relation to the management of acutely injured cattle?

________________________________________________________________

________________________________________________________________

________________________________________________________________

________________________________________________________________
