# Supplementary material for: Management of acutely injured cattle by on farm emergency slaughter: Survey of veterinarian views
Source: Front Vet Sci. 2022 Nov 10;9:976595. doi: 10.3389/fvets.2022.976595 (PMC9686391; doi:10.3389/fvets.2022.976595)
Supplement: Supplementary file 2 [file Table_2.DOCX]

Survey on the Management of Acutely Injured Cattle PVPs

Q1
 ***A questionnaire-based survey on the management of acutely injured cattle in the Republic of Ireland.***  *Dear Colleague,*  *I am a Veterinarian working in Veterinary Public Health as a Veterinary Officer with Mayo County Council.  I am conducting a project with Associate Professor Alison Hanlon, UCD School of Veterinary Medicine, Assistant Professor Flavia Santos, UCD School of Psychology and Dr. Aideen McKevitt, UCD School of Agriculture and Food Science. The survey should be answered by Private Veterinary Practitioners.*  *The survey focuses on the management of acutely injured cattle in the Republic of Ireland. An acute injury is an injury that is severe, causes acute pain, has a sudden onset, is usually associated with a traumatic event and is commonly locomotory. The overall aim of the project is to determine how acutely injured cattle are managed in Ireland. The survey considers three areas:*  *1. The methods used by PVPs' to manage acutely injured cattle.* *2. The PVPs' opinions and experience in relation to on farm emergency slaughter.* *3. The PVPs' opinions on the rules and policies in relation to on farm emergency slaughter.*
 *All data will be anonymised and stored securely in compliance with UCD rules and regulations.*  *The survey will take approximately 10 - 12 minutes and you must consent to participate in the survey.*  *The survey closes on 21st May.*  *Thanking you,*  *Yours sincerely,* *Paul McDermott, MVB, MSc (VPH), MRCVS.* *paul.mc-dermott.1@ucdconnect.ie*

- Yes, I consent
- No, I do not consent

|  |
| --- |

Q2 What age are you?

________________________________________________________________

Q3 Gender

- Male
- Female
- Other

|  |
| --- |

Q4 How many years have you been qualified?

________________________________________________________________

Q5 Where did you qualify?

- Republic of Ireland
- UK
- Other (please specify) ________________________________________________

Q6 The post-graduate qualifications that I have undertaken are (please specify)

- Graduate Cert Small Animal Medicine
- Graduate Cert Dairy Health
- Graduate Cert Equine Sports Medicine
- Graduate Cert Canine Sports Medicine
- Cert VPH
- Diploma VPH
- MSc VPH
- MVM
- Other (please specify) ________________________________________________

Q7 The main area of **expertise** I work in is (select only one)

- Food Animal (please specify species)
- Companion Animal
- Equine
- Other (please specify) ________________________________________________

Q8 **The number** of PVPs in the practice I work in are (numeric value)

________________________________________________________________

Q9 Are you a **practice** partner/assistant?

- Partner
- Assistant

Q10 The **jurisdictions** I have worked in are (tick all that apply)

- Republic of Ireland
- UK
- Other (please specify) ________________________________________________

Q11 The **practice** I work in operates in ROI/NI/Both

- Republic of Ireland
- Northern Ireland
- Both

Q12 I work as a **TVI** in a slaughterhouse

- Yes
- No

Q13 **Acutely Injured Statistics**

*On-farm emergency slaughter (OFES) refers to the on-farm slaughter of healthy cattle that has suffered an accident.*
*Casualty Slaughter refers to the slaughter of an injured cattle that has been deemed fit for transport to the abattoir under Veterinary Certification.*
 
 The number of acutely injured cattle that I had to perform casualty **OFES** on in 2020 was

________________________________________________________________

Q14  The number of acutely injured cattle that I had to certify for **casualty slaughter** on in 2020 was

________________________________________________________________

________________________________________________________________

________________________________________________________________

________________________________________________________________

________________________________________________________________

Q15 The number of acutely injured cattle that I had to perform **euthanasia** on in 2020 was

________________________________________________________________

________________________________________________________________

________________________________________________________________

________________________________________________________________

________________________________________________________________

Q16 The **nature of the injury** of the last three acutely injured cattle that I certified for **OFES** was

- 1 ________________________________________________
- 2 ________________________________________________
- 3 ________________________________________________

Q17 The **nature of the injury** of the last three acutely injured cattle that I certified for **casualty slaughter** was

- 1 ________________________________________________
- 2 ________________________________________________
- 3 ________________________________________________

Q18 The **nature** of the injury of the last three acutely injured cattle that I had to perform **euthanasia** on was

- 1 (10) ________________________________________________
- 2 (11) ________________________________________________
- 3 (12) ________________________________________________

Q19 **The number** **of abattoirs**I am aware of within a **100 Km radius** of my practice that provide the service of **OFES** is (numeric value)

________________________________________________________________

Q20 **My knowledge about**the regulations regarding the procedure of **OFES** on a scale of 0-10
 where **0 is my knowledge is extremely limited** and **10 is I am very knowledgeable** is**,**

|  | 0 | 1 | 2 | 3 | 4 | 5 | 6 | 7 | 8 | 9 | 10 |
| --- | --- | --- | --- | --- | --- | --- | --- | --- | --- | --- | --- |

| Number () | 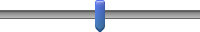 |
| --- | --- |

Q21 My **knowledge about** the management of acutely injured cattle is informed by (tick all that apply)

- Guidelines
- Regulations
- Other PVPs
- Professional Veterinary organisation
- Others (specify) (5) ________________________________________________

Q22 **T**he **time frame** for managing cattle with an acute injury **should not exceed**

- 12hrs
- 24hrs
- 48hrs
- Other (specify) ________________________________________________

Q23  **I  consult** with the following when certifying cattle for **OFES** (tick all that apply)

- Official Veterinarians (OVs) at abattoirs
- Abattoir owners
- District Veterinary Office
- Other PVPs
- I don't consult
- Other (specify) ________________________________________________

Q24 **My experience** about  the procedure known as **OFES** on a scale of 0-10 is,
 where **0 is extremely bad and 10 is extremely good**

|  | 0 | 1 | 2 | 3 | 4 | 5 | 6 | 7 | 8 | 9 | 10 |
| --- | --- | --- | --- | --- | --- | --- | --- | --- | --- | --- | --- |

| Number () | 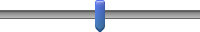 |
| --- | --- |

Q25 **My** **opinion** about the procedure known as **OFES** on a scale of 0-10 is,  
 where **0 is extremely negative** and **10 is extremely positive**

|  | 0 | 1 | 2 | 3 | 4 | 5 | 6 | 7 | 8 | 9 | 10 |
| --- | --- | --- | --- | --- | --- | --- | --- | --- | --- | --- | --- |

| Number () | 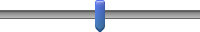 |
| --- | --- |

Q26 I would like to see OFES as a method for dealing with acutely injured cattle available nationwide

- Yes
- No

Q27 The three **positive aspects** **of OFES**, in relation to **animal welfare**, as a procedure for dealing with acutely injured cattle are

- 1 ________________________________________________
- 2 ________________________________________________
- 3 ________________________________________________

Q28 The three **negative aspects** of OFES, in relation to **animal welfare**, as a procedure for dealing with acutely injured cattle are

- 1 ________________________________________________
- 2 ________________________________________________
- 3 ________________________________________________

Q29 I would recommend the following three **changes** to the current **OFES** procedure

- 1 ________________________________________________
- 2 ________________________________________________
- 3 ________________________________________________

Q30 What matters have you **discussed with other PVPs** about the management of acutely injured cattle

________________________________________________________________

________________________________________________________________

________________________________________________________________

________________________________________________________________

Q31 What matters have you discussed with Official Veterinarians about the management of acutely injured cattle

________________________________________________________________

________________________________________________________________

________________________________________________________________

________________________________________________________________

________________________________________________________________

Q32 In your opinion are the policy/rules in the Standard Operating Policy that enable the procedure of OFES are on a scale of 0-10,
 where 0 is very restrictive and 10 is not restrictive

|  | 0 | 1 | 2 | 3 | 4 | 5 | 6 | 7 | 8 | 9 | 10 |
| --- | --- | --- | --- | --- | --- | --- | --- | --- | --- | --- | --- |

| Number () | 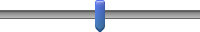 |
| --- | --- |

Q33 If rules are too **restrictive** please give three examples

- 1 ________________________________________________
- 2 ________________________________________________
- 3 ________________________________________________

Q34 I work in a practice that has a **practice policy** in relation to OFES

- Yes
- No

Q35 Indicate on a scale of 0 - 10 if the practice policy is **in favour** of using OFES as a method of managing acutely injured cattle, 
 **where 0 is extremely not in favour and 10 is extremely in favour**

|  | 0 | 1 | 2 | 3 | 4 | 5 | 6 | 7 | 8 | 9 | 10 |
| --- | --- | --- | --- | --- | --- | --- | --- | --- | --- | --- | --- |

| Number () | 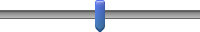 |
| --- | --- |

Q36  My **decision making** in relation to the management of acutely injured cattle is influenced by (tick all that apply)

- Practice Policy
- Other PVPs
- OVs
- Farmers
- Abattoir owners
- Veterinary Organisations
- Other (specify) ________________________________________________

Q37 Does OFES have any **unrealised potential?**

- Yes
- No

Q38 **If yes**, can you outline three ways that **this potential** may be realised so as to encourage wider adoption of the procedure

- 1 ________________________________________________
- 2 ________________________________________________
- 3 ________________________________________________

Q39 Is there **anything else** you would like to add in relation to the management of acutely injured cattle?

________________________________________________________________

________________________________________________________________

________________________________________________________________

________________________________________________________________

________________________________________________________________
